# The bud tip is the cellular hot spot of protein secretion in yeasts

**DOI:** 10.1007/s00253-016-7674-6

**Published:** 2016-06-23

**Authors:** Verena Puxbaum, Brigitte Gasser, Diethard Mattanovich

**Affiliations:** 1 Department of Biotechnology, University of Natural Resources and Life Sciences, Muthgasse 18, 1190 Vienna, Austria; 2 Austrian Centre of Industrial Biotechnology (ACIB GmbH), Vienna, Austria

**Keywords:** Protein secretion, *Pichia pastoris*, Recombinant protein, Fluorescence microscopy, Subcellular localization, Bud

## Abstract

Yeasts are valuable hosts for recombinant protein production. Among them, *Pichia pastoris* is frequently used for production of secreted proteins, and much effort was made to improve the secretion efficiency of this expression platform. However, the knowledge on the secretion machinery is mainly based on studies in *Saccharomyces cerevisiae*. Therefore, it is of great interest for targeted improvement of the system to learn more about the secretion process in *P. pastoris*. Using human serum albumin, a protein which is produced in high quantities in *P. pastoris*, we show here the secretion pathway of this protein. During passage of the secretory route, the recombinant protein is mainly localized in the endoplasmic reticulum (ER) and in COPII vesicles, and is inherited to the daughter cell via the perinuclear ER. The final release to the cell exterior occurs at the bud, initiating at the bud tip and later spreading over the entire bud surface. The same polarized secretion pattern was observed for a recombinant antibody light chain and the native secretory protein Epx1 of *P. pastoris*. Clarifying the point of release of secretory proteins will have major impact on engineering the secretory pathway of *P. pastoris* and other budding yeasts.

**Electronic supplementary material:**

The online version of this article (doi:10.1007/s00253-016-7674-6) contains supplementary material, which is available to authorized users.

## Introduction

The yeast *Pichia pastoris* (*Komagataella* spp.) is an important host for recombinant protein production which is successfully used for the production of various pharmaceutical proteins and technical enzymes (Gasser et al. 2013; Weinacker et al. 2013; Ahmad et al. 2014; Spohner et al. 2015). The method of choice for protein production in yeast is secretion of the product into the culture supernatant for convenient product recovery and purification, and for eventual product glycosylation. However, the complexity of the secretory pathway introduces some obstacles to protein production. On the secretory pathway, the protein is folded in the endoplasmic reticulum (ER); after successful folding, it is transported to the Golgi where it is packed in secretory vesicles which then finally fuse with the plasma membrane. All these mentioned steps can be limiting factors for the overproduction of foreign proteins in large amounts. For the ER, this is supported by a significant increase in secretion efficiency for various products when co-overexpressed with chaperones (Gasser et al. 2006; Inan et al. 2006; Delic et al. 2012). For a recent review see Puxbaum et al. (2015). Furthermore, there is evidence that the nature of the protein influences the secretion ability, resulting in varying titers depending on the respective secreted protein. Human serum albumin, for example, is secreted in grams per liter amounts (Kobayashi et al. 2000) whereas heterodimeric antibody fragments are often found only in milligrams per liter (Gasser et al. 2006). Impaired folding may lead to degradation of a large fraction of recombinant protein via the endoplasmic reticulum-associated protein degradation pathway (ERAD) or later in the vacuole (recently reviewed in Puxbaum et al. 2015). It was shown for different recombinant proteins that inhibiting the degradation routes (ERAD and vacuole) improved protein production in yeast (Idiris et al. 2010; Pfeffer et al. 2012).

The final step of constitutive secretion is the exocytosis event when the secretory vesicles which have been released from the trans-Golgi network fuse with the plasma membrane, involving a cluster of proteins, the so-called exocyst-complex (Guo et al. 1999; He et al. 2007a,b; reviewed in Hsu et al. 2004). The crucial importance of these proteins for secretion was elegantly shown for Sec1, a protein that is involved in fusion of the secretory vesicles with the exocyst. Using a temperature-sensitive Sec1 mutant which is not capable in fusion with the exocyst resulted in an accumulation of secretory vesicles in the cells. After return of the cells to permissive temperature, the number of vesicles was reduced and the cargo acid phosphatase was secreted (Novick and Schekman 1979). Concerning the point of delivery of secretory vesicles, it was demonstrated that acid phosphatase and other cell wall-bound components like mannan are incorporated at the bud membrane (Farkas et al. 1974; Field and Schekman 1980).

All this information is mainly based on studies of *Saccharomyces cerevisiae*; however, there is no detailed information about secretion and exocytosis in *P. pastoris*. In general, the secretion model of *S. cerevisiae* is believed to be also true for *P. pastoris*. For a review on genetic similarity and differences of the secretory pathway see Delic et al. (2013). Anyhow, there are known differences between these two species, as for example in the Golgi apparatus. The structure and distribution of the Golgi in *P. pastoris* cells is ordered in stacks and is therefore more similar to mammalian cells (Rossanese et al. 1999).

For improving recombinant protein production in *P. pastoris* and other yeasts, it is important to understand the secretion processes. Using different microscopy methods, the secretion of recombinant and native proteins was studied in detail to characterize the intracellular passage and the release from the cell.

## Material and methods

### Yeast strains and plasmids

*P. pastoris* CBS7435 was transformed with the human serum albumin (HSA) gene expressed under the strong, constitutive GAP (glyceraldehyde 3-phosphate dehydrogenase) promoter. HSA was secreted using its native secretion leader. The sequence was codon-optimized for *P. pastoris* (GenBank accession number: KX000915). HSA was fused to oxGFP (Costantini et al. 2015) or mCherry (Shu et al. 2006) by introducing a *Not*I restriction site resulting in three alanine residues between the two proteins. The plasmid was integrated in the *AOX1* terminator region and selection was performed using either the zeocin or the kanamycin/G-418 resistance cassette. For surface display immunostaining, the gene encoding the C-terminal membrane bound fragment of *S. cerevisiae* α-agglutinin (*SAG1*, Stadlmayr et al. 2010) was fused to the C-terminus of HSA or the light chain of IgG against hen egg lysozyme (HyHEL, Heiss et al. 2015) and expressed under the repressible P_G1_ promoter (Prielhofer et al. 2013). For cell surface detection of a native *P. pastoris* protein, the *EPX1* gene fused to a c-myc epitope sequence followed by the α-agglutinin sequence at the 3′-terminus was expressed under control of the P_G1_ promoter. Vector maps of used plasmids are shown in the supplementary Fig. S1. The plasmids Sec13-DsRed and Sec7-3xGFP were kindly provided by B. Glick, University of Chicago.

### Cultivation

For screening of best clones, 2 mL YPD (per liter: 10 g yeast extract, 20 g peptone, 20 g glucose) containing either 25 μg mL^−1^ zeocin or 500 μg mL^−1^ G-418 in 24-deep well plates were inoculated with single colonies and incubated shaking at 280 rpm with 25 mm amplitude on 25 °C over night. The next day, aliquots corresponding to OD_600_ = 0.1 were transferred into 24-deep well plates containing 2 mL M2 minimal medium (20 g citric acid, 3.15 g (NH_4_)_2_HPO_4_, 0.03 g CaCl_2_.2H_2_O, 0.8 g KCl, 0.5 g MgSO_4_.7H_2_O, 2 mL biotin (0.2 g L^−1^), 1.5 mL trace salts stock solution for 1 L of M2 minimal medium. The pH was set to 5.0 with 5 M KOH solution. The trace salts stock solution contained per liter: 6.0 g CuSO_4._5H_2_O, 0.08 g NaI, 3.0 g MnSO_4_. H_2_O, 0.2 g Na_2_MoO_4_.2H_2_O, 0.02 g H_3_BO_3_, 0.5 g CoCl_2_, 20.0 g ZnCl_2_, 5.0 g FeSO_4_.7H_2_O and 5.0 mL H_2_SO_4_ (95–98 % *w*/*w*), Delic et al. 2012) + 2 % glucose and cultivated at 25 °C for 48 h shaking at 180 rpm with 25 mm amplitude. The cultures were fed with 1 % glucose every 12 h. Cultivation conditions for expression under the P_G1_ are described in the section “Surface display immunostaining”.

### Immunoblot

Cells were grown in minimal medium for 48 h starting with an OD_600_ of 0.1. The cultures were centrifuged at 3000 rpm for 3 min, the supernatant was kept for further analysis and the cell pellet was washed and lysed. For lysis, the pellet was resuspended in 500 μL lysis buffer (20 mL 1 × PBS, one tablet SIGMA*FAST* protein inhibitor tablets (Sigma-Aldrich, St. Louis, MO, USA), 2 % Triton X-100) and 250 μL glass beads (0.5 mm) were added. Cells were lysed for 3 × 20 s at level 6 with the FastPrep cell disruptor (MP Biomedicals, Santa Ana, CA, USA). Afterwards, 10 μL of 1 M SDS solution was added and the samples were boiled for 10 min. Finally, the samples were centrifuged for 5 min at 13,000 rpm and the supernatant was used for SDS-PAGE. Equal volumes of lysate and culture supernatant were mixed with NuPAGE LDS sample buffer and reducing agent (Life Technologies, Carlsbad, CA, USA) and heated to 70 °C for 5 min. The samples were applied on a NuPAGE 12 % Bis-Tris Gel (Life Technologies, Carlsbad, CA, USA) and blotted on a nitrocellulose membrane. The blots were blocked in 2 % BSA in PBS Tween (0.05 %) for at least 2 h. HSA-oxGFP was detected by incubation with rabbit anti-GFP antibodies (Abcam, Cambridge, UK, 1:5000) for 1.5 h at RT followed by washing steps and incubation for 1 h with secondary anti-rabbit antibodies conjugated with HRP (Sigma-Aldrich, St. Louis, MO, USA, 1:16,000). For detection, Pierce ECL solution was used and the bands were detected with a LumiImager (Boehringer Mannheim, Mannheim, Germany).

### Immunofluorescence microscopy

HSA-expressing *P. pastoris* was cultivated and stained according to Delic et al. (2012). As primary antibodies, goat anti-human albumin (Bethyl Laboratories, Montgomery, TX, USA, 1:1000) and mouse monoclonal anti-HDEL antibodies (clone 2E7, Santa Cruz Biotechnology, Heidelberg, Germany, 1:100) were used. The applied secondary antibodies were donkey anti-goat IgG Alexa Fluor 488 (1:200) and donkey anti-mouse IgG Alexa Fluor 647 (1:100) antibodies (Molecular Probes, Eugene, OR, USA). The cells were viewed in a Leica SP5 confocal microscope using a HCX PL APO CS 63.0 × 1.30 NA glycerol objective and 488 and 633 nm lasers. Images were processed using ImageJ (Rasband W.S. ImageJ, U.S. National Institutes of Health, Bethesda, MD, USA, http://imagej.nih.gov/ij/, 1997–2016).

### 4D video microscopy

Cells were grown in 10 mL M2 minimal medium + 2 % glucose for approximately 24 h shaking at 180 rpm with 25 mm amplitude at 25 °C to an OD_600_ of about 1. For short movies, cell suspension was put directly on a glass slide, covered with a cover slip, and monitored every 2 min performing a z-stack covering the entire cell (*z* = 28) on a Leica DMI6000 epifluorescence microscope. For long-term movies to view a complete cell cycle event, cells were put on an agarose pad. Four percent low-melting agarose was melted in M2 minimal medium at 90 °C for approx. 15 min. One hundred microliters of liquid agarose was put in the middle of a glass slide which was surrounded by tape and covered with a second glass slide. After 10 min, the second slide was taken off and 20 μL of the cell suspension (OD_600_ ~ 1) was put on the agarose pad and covered with a 24 × 50 mm cover slip. The cells were monitored on a Leica SP5 confocal microscope for 3 h using a HCX PL APO CS 63 × 1.2NA water objective and a 488-nm laser and bright field. A z-stack (22 steps at step size of 0.3 μm) was made every 5 min. Images were processed using ImageJ (Rasband W.S. ImageJ, U.S. National Institutes of Health, Bethesda, MD, USA, http://imagej.nih.gov/ij/, 1997–2016).

### Surface display immunostaining

Fifty microliters pre-culture was pipetted into 50 mL M2 minimal medium + 2 % glucose and incubated at 25 °C for 24 h shaking at 180 rpm with 25 mm amplitude. Cells were washed with 10 mL M2 minimal medium without glucose and three Erlenmeyer shake flasks with 10 mL M2 minimal medium without glucose were inoculated in parallel at an OD_600_ of 1 and one FeedBead disk (slow glucose release, diameter 12 mm, Kuhner shaker, Birsfelden, Switzerland) was added to each flask. Cultures were incubated at 25 °C and after 1, 2, and 3 h, samples were taken by harvesting one Erlenmeyer flask. Therefore, 8 mL of cell suspension was centrifuged at 2000 rpm for 3 min. Cells were resupended in 5 mL of 4 % paraformaldehyde in PBS + 1 mM MgCl_2_ and incubated at RT for 1 h on an end-over-end mixer. After incubation, cells were washed two times with 5 mL 100 mM potassium phosphate buffer (pH = 6.5) + 1 mM MgCl_2_ and finally resuspended in 1 × PBS + 0.5 % BSA to a final OD_600_ of 10. All samples were kept on ice until the last sample was fixed. One milliliter of each sample was transferred to a 1.5 mL centrifugation tube and centrifuged for 3 min at 2500 rpm. The cells were resuspended in 1 mL of antibody solution (either goat anti-human albumin (Bethyl Laboratories, Montgomery, TX, USA, 1:500) or mouse monoclonal anti-myc antibodies (clone 4A6, EMD Millipore, Darmstadt, Germany, 1:100)) and incubated 1 h end-over-end at RT. The cells were washed 3 times with 1 × PBS + 0.5 % BSA and incubated with 1 mL of the secondary antibody solution (donkey anti-goat IgG Alexa Fluor 488 or donkey anti-mouse IgG Alexa Fluor 488, 1:200, Molecular probes, Eugene, OR, USA) for 45 min end-over-end at RT in the dark. Cells were washed again three times with 1 × PBS + 0.5 % BSA and were finally resuspended in 200 μL 1 × PBS. The cells were put on a microscope slide, covered with a cover slip, and viewed in a Leica SP5 confocal microscope using a HCX PL APO CS 63 × 1.2NA water objective and a 488 nm laser. Images were processed using ImageJ (Rasband W.S. ImageJ, U.S. National Institutes of Health, Bethesda, MD, USA, http://imagej.nih.gov/ij/, 1997–2016).

## Results

### Recombinant human serum albumin is located to ER and COPII vesicles

**Fig. 1:**
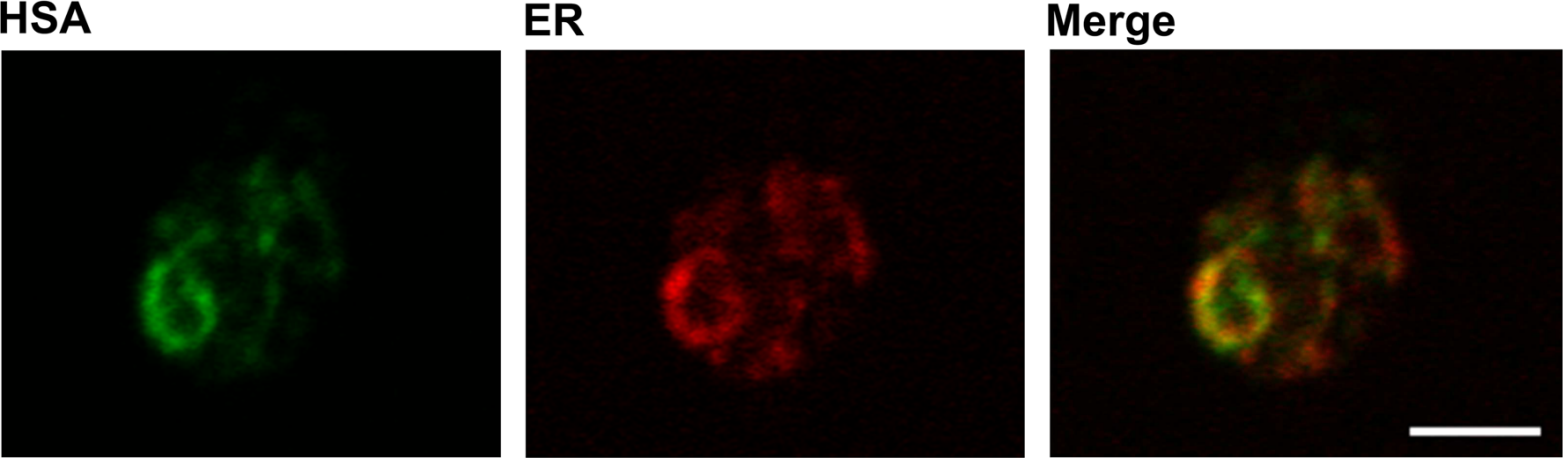
Subcellular localization of HSA in the ER. *P. pastoris* expressing HSA was immunostained with a goat anti-HSA antibody and a fluorescently labeled secondary antibody (donkey anti-goat IgG Alexa Fluor 488) to detect HSA (*left*) and a mouse anti-HDEL antibody and a fluorescently labeled secondary antibody (donkey anti-mouse IgG Alexa Fluor 555) to detect the ER (*middle*). The two fluorescence images were merged (*right*) and revealed co-localization of the two proteins. The cells were viewed in a confocal laser scanning microscope. *Bar*, 3 μm

As a major model protein, human serum albumin (HSA), a protein which can be produced in high quantities in *P. pastoris* (Kobayashi et al. 2000), was used. Therefore, *P. pastoris* codon-optimized HSA was expressed under the control of the constitutive glyceraldehyde 3-phosphate dehydrogenase (GAP) promoter using the HSA native secretion leader in the *P. pastoris* strain CBS7435. Co-stainings with intracellular markers revealed an intense perinuclear endoplasmic reticulum pattern for HSA in the cells (Fig. 1).

**Fig. 2:**
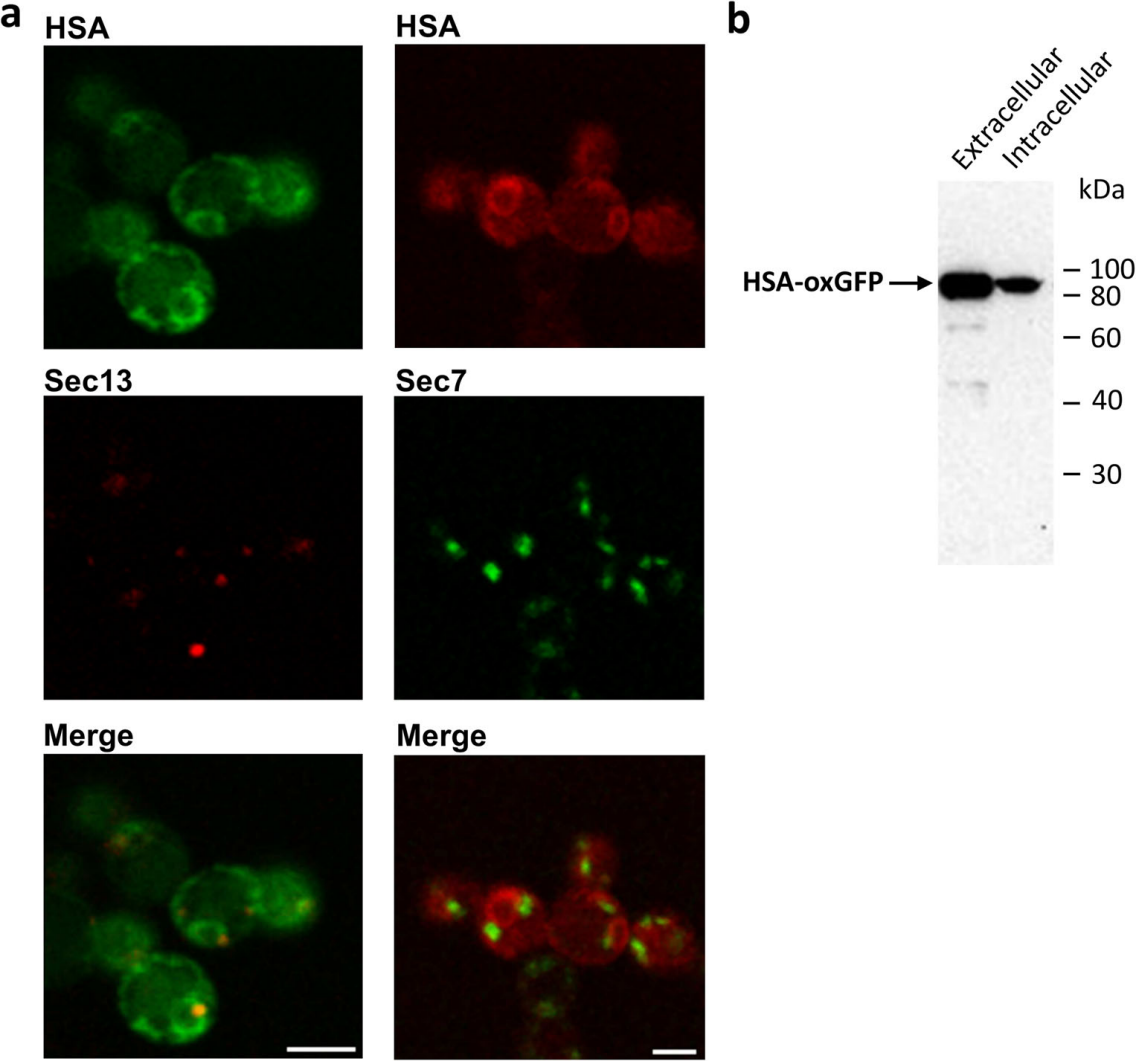
Fluorescent fusion protein HSA-oxGFP is localized in COPII vesicles, but not in the Golgi. **a** *P. pastoris* expressing HSA-oxGFP and the COPII vesicle marker Sec13-DsRed (*left panel*) and HSA-mCherry and the Golgi marker Sec7-3xGFP (*right panel*). Cells were viewed in a confocal laser scanning microscope. *Bars*, 3 μm. **b** Immunoblot of HSA-oxGFP using rabbit anti-GFP IgG and secondary anti-rabbit IgG conjugated with HRP. After cultivation of 48 h, cells were harvested, lysed, and lysate as well as supernatant were applied on the gel and blotted on a nitrocellulose membrane. The positions of selected molecular mass standards are indicated

In order to perform live cell imaging, an HSA-GFP fusion protein was created. Hence, HSA was C-terminally fused to oxGFP (a GFP variant with mutated cysteines, especially suitable for visualizing proteins in the secretory pathway, kindly provided by E. Snapp, Albert Einstein College of Medicine, New York) under the control of the GAP-promoter and integrated by homologous recombination in the *AOX1*-terminator region. This fusion protein showed the previously observed ER pattern as well. The cells were co-transformed with the tER/COPII marker Sec13-DsRed, and a clear co-localization of the proteins was noticed (Fig. 2a). As Sec13-DsRed concentrates at tER sites, which are ER subdomains specialized for COPII vesicle budding (Rossanese et al. 1999), this co-localization confirms the presence of HSA-oxGFP in the ER. Co-expression of HSA-mCherry and the late Golgi marker Sec7-3xGFP revealed no co-localization of the two proteins, probably due to a short residence time of HSA in the Golgi apparatus (Fig. 2a). An immunoblot of the cell lysate and the supernatant was performed to verify if the intact fusion protein HSA-oxGFP is synthesized and secreted. HSA-oxGFP appeared at the expected size of 93.5 kDa and was secreted into the culture medium. No free GFP at the size of 27 kDa was detected in the cells, indicating that the GFP signal derives from intact fusion protein (Fig. 2b). This is of great importance to assure that only the fusion protein is detected during live cell monitoring and artifacts due to free GFP are avoided.

### Recombinant protein is inherited to the daughter cell via ER separation during cell division

**Fig. 3:**
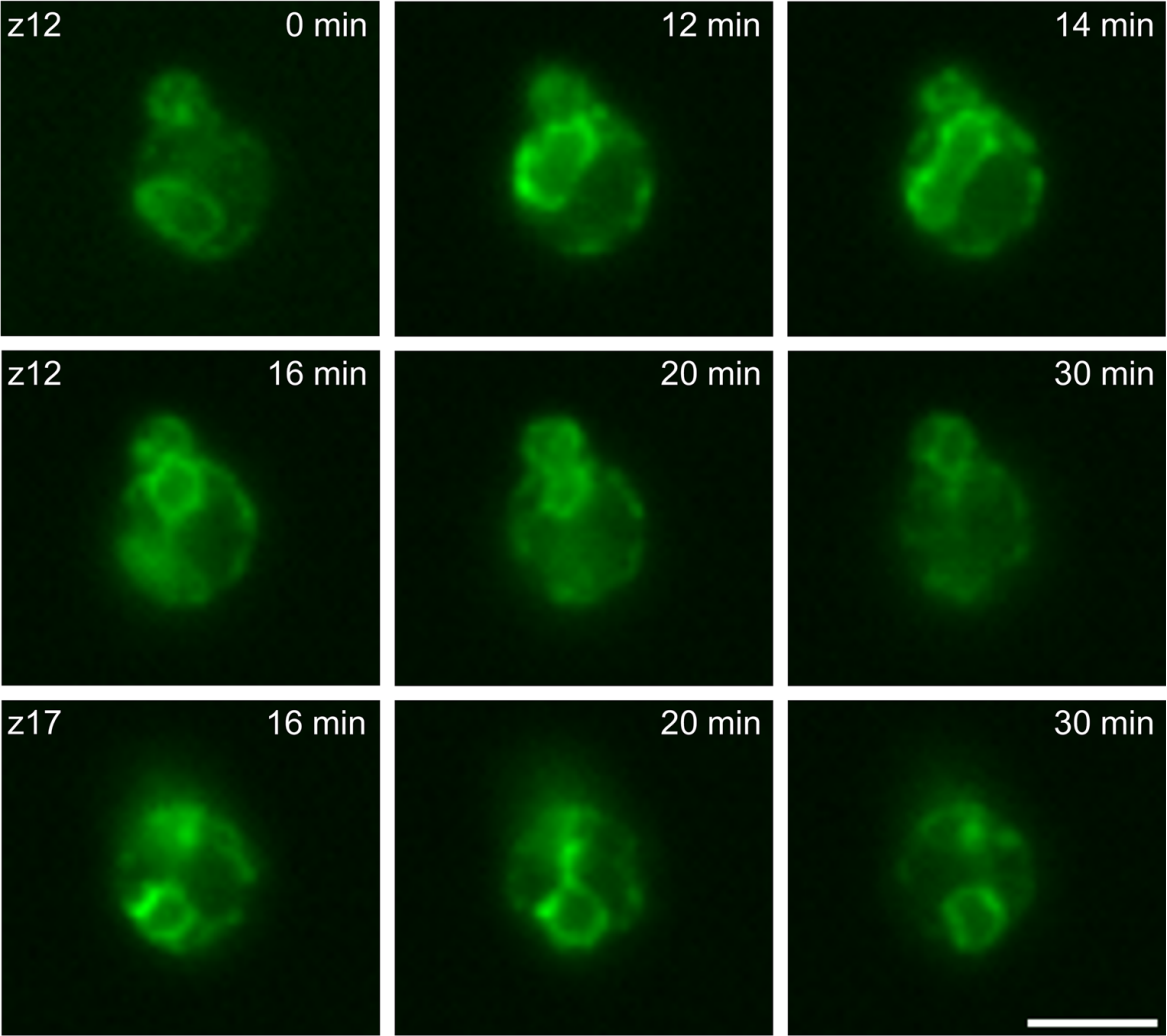
Time-lapse microscopy of *P. pastoris* expressing HSA-oxGFP. Cells were directly put on a slide and monitored in an epifluorescence microscope. Every 2 min, a z-stack covering the entire cell was made. Here, layers 12 and 17 are shown at the indicated time points. *Bar*, 3 μm

For live cell microscopy, *P. pastoris* which constitutively expressed HSA-oxGFP was monitored in an epifluorescence microscope. A time series was taken for 30 min, meaning that every 2 min a z-stack covering the whole cell was carried out. Applying this technique, we were able to observe the inheritance of HSA, which was localized in the ER, to the bud during cell division. The phenomenon of ER inheritance to the bud in *S. cerevisiae* has been reported by Prinz et al. (2000). Here, we show that recombinant protein enters the bud by inheritance of the perinuclear ER, the organelle in which HSA was mainly found in the cells. In Fig. 3, the most relevant time points of the time-lapse experiment are presented. At the beginning, the typical perinuclear ER ring is seen, the ER then moves near to the bud (12 min), and the separation of the nucleus together with the perinuclear ER is starting (14 min). After 16 min, nucleus and ER are already separated and in z 12 the final movement to the bud is seen whereas the remaining nucleus plus perinuclear ER (z 17) in the mother cell moved back to the starting position in the cell near the plasma membrane. The continuous movies (z 12 and z 17) are found in the Online Resources 1 and 2.

### Recombinant protein is secreted to the cell exterior at the bud tip

**Fig. 4:**
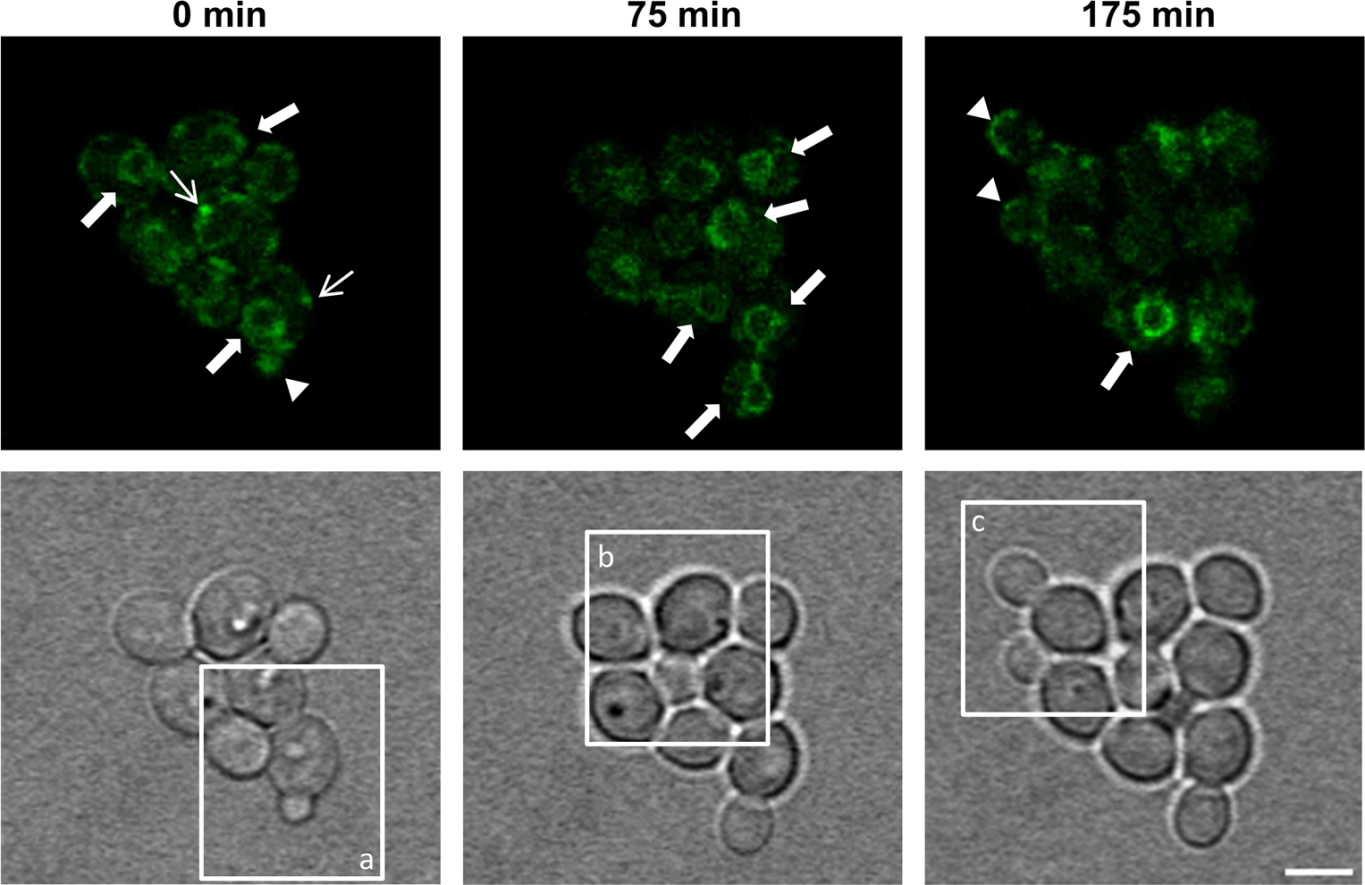
Long-term time-lapse microscopy of *P. pastoris* expressing HSA-oxGFP. Cells were put on a 4 % agarose pad and monitored in a confocal microscope. Every 5 min, a stack covering the entire cell was made. Here, the time points of layer 13 displaying the most important findings are shown. *Upper panel*: fluorescence images at indicated time points, *lower panel*: bright field images at respective time points. Selected areas indicated as *a*, *b*, and *c* are shown in Fig. 5. *Bold arrows* indicate the localization of HSA-oxGFP in the ER, *thin arrows* point to tER sites and the bud tips are highlighted by *arrowheads*. *Bar*, 3 μm

**Fig. 5:**
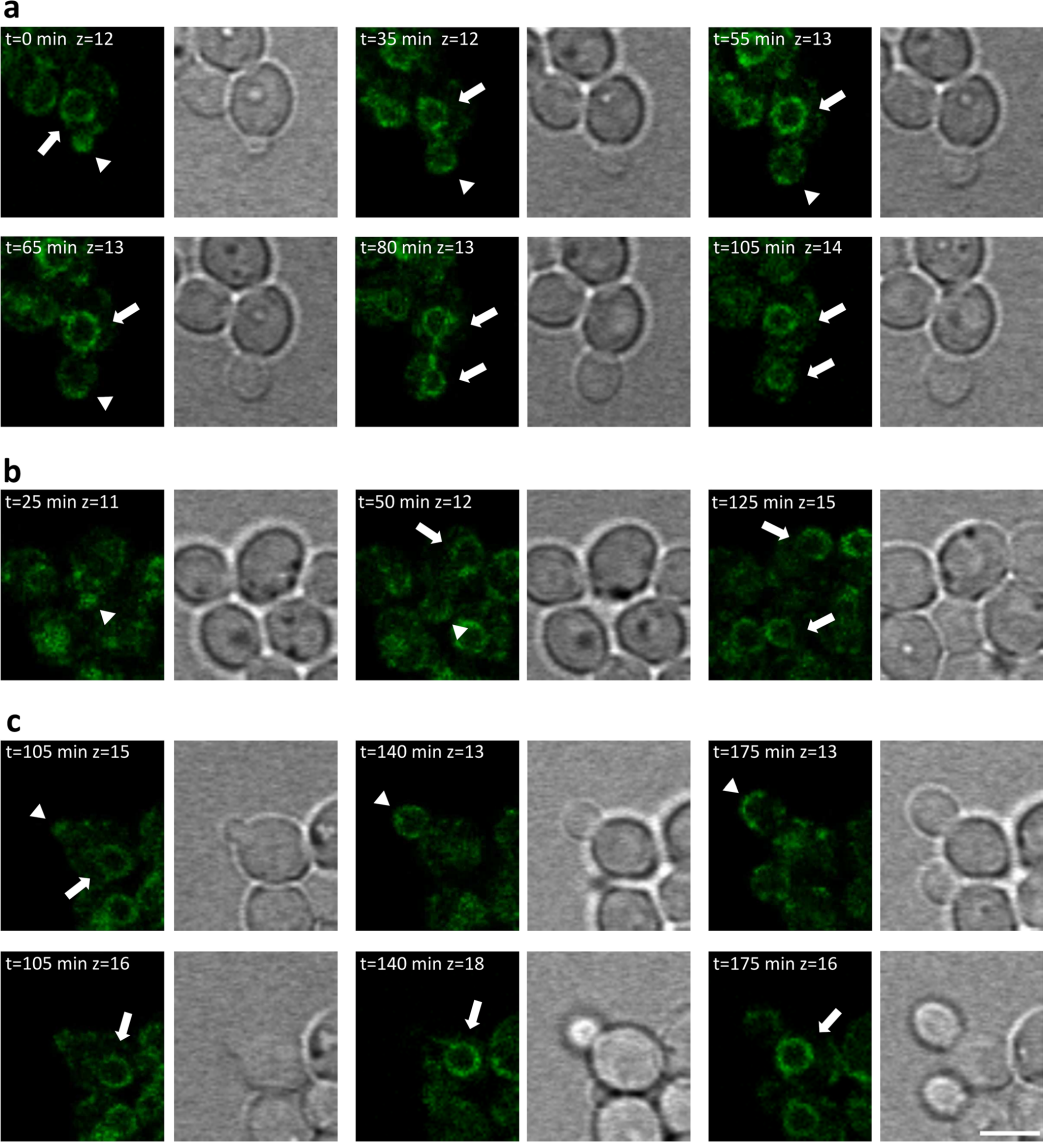
Selection of individual cells (marked as *a*, *b*, *c*) from long-term time lapse microscopy images shown in Fig. 4. Several time points (indicated in *minutes*) and representative layers (*z*) to follow secretion events in single cells in a chronological order are depicted. Fluorescence images as well as the respective bright field images are shown. *Bold arrows* indicate the localization of HSA-oxGFP in the ER and bud tips are highlighted by *arrowheads*. *Bar*, 3 μm

Growing and dividing cells which expressed the HSA-oxGFP fusion protein were tracked alive for a complete cell cycle event. Therefore, cells were placed on an agarose pad and images were taken in 5 min intervals on a confocal microscope. The cells were monitored for 3 h (Online Resources 3 and 4). In almost all cells, HSA-oxGFP was found in the ER, at tER sites, and in the bud, indicated by respective arrows. Images of the most informative time points in one z-layer (*z* = 13) are shown in Fig. 4. Recombinant fusion protein was observed in the ER (bold arrow), at tER sites (thin arrow), and also at the bud tip (arrowhead). As expected from the previous experiments, HSA-oxGFP was localized in the ER of mother cells while the cell surface was primarily stained at the tip of growing buds. In the middle panel also the event of inheritance of HSA to the daughter cell, as already described above, was noticed again (bold arrows). At the last time point shown in Fig. 4 (175 min), a clear signal at the tips of two growing buds was visible (arrowheads) as well as a prominent ER pattern in another cell. To display the secretory events in chronological sequence, single cells in their most representative z-layers are depicted in Fig. 5. The individual frames chosen for Fig. 5 are indicated in the bright-field images in Fig. 4. In Fig. 5a, HSA-oxGFP is visible at the emerging bud, the ER and at tER sites at the beginning of the time series (*t* = 0 min). In the following 65 min, the bud was growing and the signal at the bud tip remained. At the time point of 80 min, the inherited ER became visible in the bud and from this time on the signal at the bud tip had vanished. This pattern remained until the end of the time series. In Fig. 5b, another cell is monitored where the bud and the HSA signal in the bud started evolving 25 min after starting the time series. Also in this cell, the signal in the bud tip remained visible (*t* = 50 min) until an ER pattern in the bud emerged and tip staining disappeared (*t* = 125 min). In Fig. 5c, the same time points in different layers are shown. The upper row shows the signal at the bud tip of the growing bud, whereas the lower row displays the layer of the ER pattern, which was only detected in the mother cell. This is in accordance with the results in Fig. 5a, b as there was still a signal in the bud tip at the latest time point (*t* = 175 min), and no inheritance of the ER had happened yet.

**Fig. 6:**
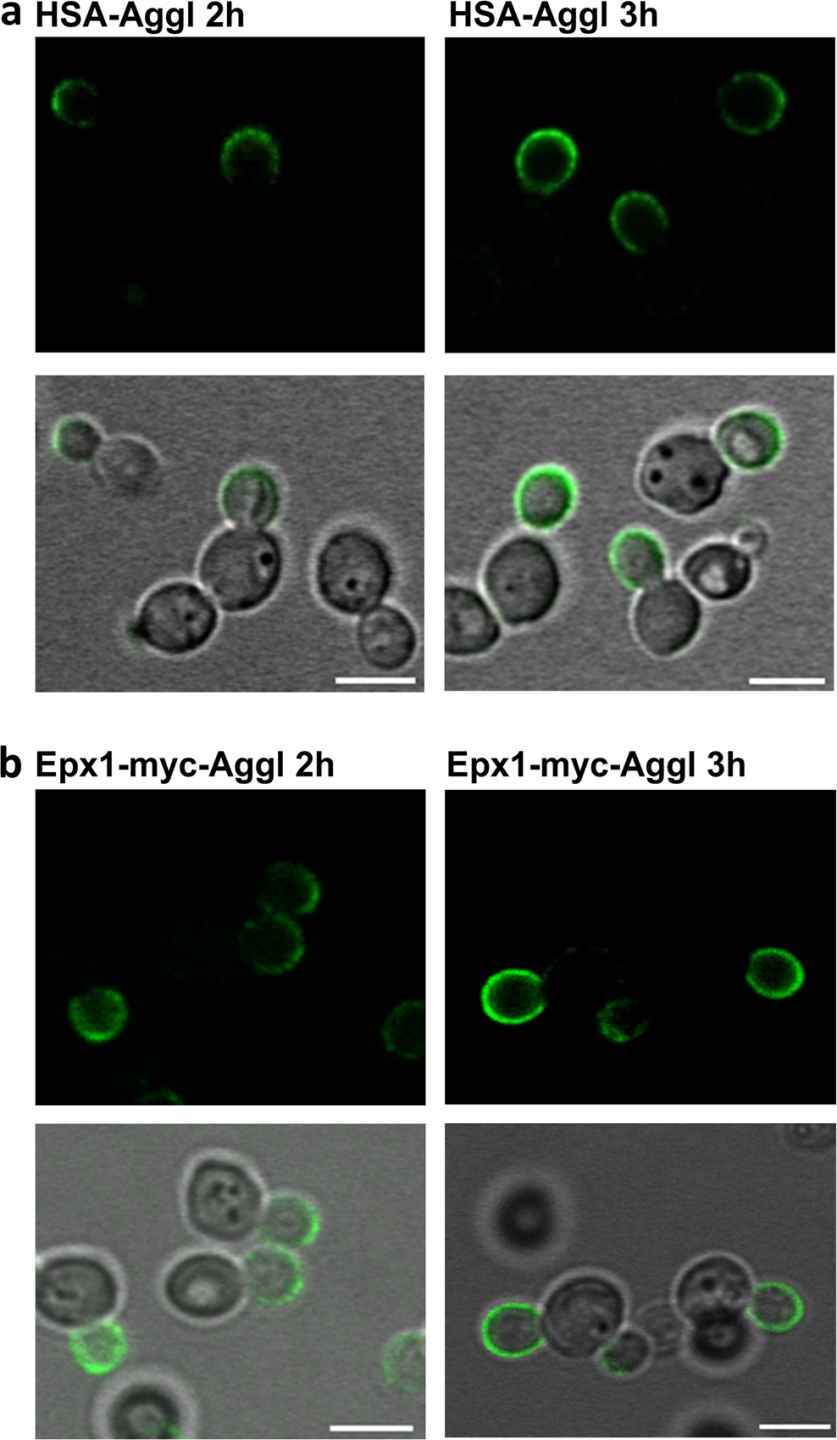
Release of secreted proteins on the cell surface. After initial growth in glucose-containing medium for 24 h, the cells were shifted to low glucose conditions. After fixation, cells were stained and viewed in a confocal laser scanning microscope. Fluorescent images as well as the overlays with bright field are shown. **a** *P. pastoris* expressing HSA-agglutinin under the P_G1_ promoter 2 and 3 h after de-repression. **b** *P. pastoris* expressing Epx1-myc-agglutinin under the P_G1_ promoter 2 and 3 h after de-repression. *Bars*, 4 μm

To verify these secretion events through the bud, the HSA gene was fused to the C-terminal cell wall anchored fragment of *S. cerevisiae SAG1* (Stadlmayr et al. 2010) and expressed under control of the repressible promoter P_G1_ (Prielhofer et al. 2013). The cells were grown in surplus of glucose and then shifted to glucose limit (achieved by cultivation with glucose feed beads) to induce HSA-agglutinin production. Due to the agglutinin anchor, the secreted protein is immobilized in the cell wall and can be detected by immunostaining. Every hour cells were fixed in 4 % paraformaldehyde followed by staining with a goat anti-HSA antibody and donkey anti-goat IgG Alexa Fluor 488 and viewed in a confocal laser scanning microscope. The first signal appeared 2 h after de-repression revealing HSA at the bud tip. Three hours after de-repression, the entire bud surface was covered with the protein (Fig. 6a). Taken together, HSA is secreted through the bud beginning at the bud tip, and as the bud grows, the protein is secreted over the entire bud surface. A further scenario could be that HSA is constantly secreted via the bud tip and as the bud grows, the anchored HSA moves from the tip to subapical parts while the actual secretion still takes place in the bud tip only. The same pattern was also observed for secreting the light chain of an anti-hen egg lysozyme antibody (HyHEL, data not shown).

### Native protein is also secreted through the bud

To verify whether secretion through the bud, as monitored for recombinant proteins, is also true for natively secreted *P. pastoris* proteins, we expressed the major secretory protein of *P. pastoris*, Epx1 (the homolog of *S. cerevisiae* Pry1, Heiss et al. 2013), in the same surface display setup. Therefore, *EPX1* with its native leader was expressed under the repressible promoter P_G1_. The protein was C-terminally fused to the c-myc epitope followed by the agglutinin cell wall anchor sequence. Again, cells were shifted to glucose-limited growth conditions followed by paraformaldehyde-fixation every hour. The cells were stained with mouse monoclonal anti-myc antibodies and donkey anti-mouse IgG Alexa Fluor 488 antibodies and viewed in a confocal microscope. Also for the native protein, a clear indication for major secretion through the bud was observed (Fig. 6b). The signal at the bud which was already fairly intense 2 h after de-repression was even more pronounced after 3 h.

## Discussion

While the single events of the secretory pathway and the underlying genes and proteins are well documented in yeast, there is only scarce information on the morphological details, especially regarding the late events where the protein passes the cell membrane and leaves the cell. Due to the high relevance of these events for the production of secreted proteins for biotechnological use, we have set out to characterize the passage of a recombinant protein through the secretory pathway of *P. pastoris* and further verified our findings with another recombinant and a native *P. pastoris* protein.

On its passage through the secretory pathway, recombinant HSA was mainly localized in the ER and COPII vesicles, but not in the Golgi. This indicates that flux of the recombinant protein through the Golgi system is probably rapid and not rate limiting, thus leading to low local steady state concentrations. Concentration in the ER is obviously higher due to a longer residence time of the protein during folding, indicating that folding is a rate-limiting step during the secretion process of recombinant HSA, and other proteins which display clear ER localization as well (Heiss et al. 2015). Signal half-life of recombinant human IgG in the ER was reported to be 1.36 h in *S. cerevisiae* (de Ruijter and Frey 2015). The benefit of co-overexpression of genes encoding for folding assisting proteins like *PDI1*, *ERO1*, and *KAR2* on the secretion rate of various recombinant proteins (reviewed in Hou et al. 2012; Delic et al. 2014) also supports the hypothesis of ER located folding as a general bottleneck in secretory production of recombinant proteins in *P. pastoris* and other yeasts.

Prinz et al. (2000) have reported that during the cell cycle of *S. cerevisiae* the ER is inherited to the bud during nuclear fission and entry to the bud. We noticed a similar phenomenon for *P. pastoris* and could demonstrate that recombinant HSA is inherited to the bud via the perinuclear ER. Thus, one main entry route of secretory protein to the bud is the transport of the nucleus with perinuclear ER into the emerging daughter cell. It remains to be determined however whether additional transport of recombinant protein via COPII vesicles to the Golgi, and secretory vesicles to the outer membrane occurs between mother cell and bud.

We could further show that in *P. pastoris* the terminal secretion event into the culture supernatant occurs through the bud tip. For the cell wall polysaccharides glucan and mannan, and the cell wall localized acid phosphatase, it was reported that newly synthesized material is incorporated into the growing buds of *S. cerevisiae* (Johnson and Gibson 1966; Farkas et al. 1974; Field and Schekman 1980). This bud site incorporation was allocated to the polarized growth of yeast, meaning that the bud is the major site of cell wall growth. However, until now, no information is available on the site where soluble cargo is released from the yeast cell. With the reported findings, we are now certain that secretion of soluble extracellular protein is happening on the secretory route via the bud. Understanding the morphological pathway of protein secretion and release from the cell is of major importance to design further improvement of this process for the production of secreted recombinant proteins.

In 2011, we showed that constitutive overexpression of the B-type cyclin *CLB2* in *P. pastoris* shifted the population towards the G2/M phase of the cell division cycle, resulting in a higher fraction of cells in the budding state. This led to a higher secretion efficiency of various recombinant proteins also at low specific growth rates, where wild-type cells usually exhibit low secretion rates (Buchetics et al. 2011; Rebnegger et al. 2014). As we showed here that the bud is the definite site of protein release from the cell, it becomes evident why a mutant that keeps more cells in budding state results in higher secretion efficiency.

Our data clearly visualize the major secretion route of secreted recombinant proteins as well as of native secretory proteins through the bud. This is a distinct indication for polarized secretion being the preferred pathway for soluble extracellular proteins in *P. pastoris*. Using several microscopic techniques, we showed that recombinant secretory protein is localized in the ER and COPII vesicles during the secretory passage in *P. pastoris*, and inherited to the bud with the perinuclear ER during the cell division cycle. Final release from the cell surface to the culture supernatant occurs at the bud tip.

## Electronic supplementary material

Fig S1 (PDF 321 kb)

Online Resources 1 (AVI 69 kb)

Online Resources 2 (AVI 70 kb)

Online Resources 3 (AVI 159 kb)

Online Resources 4 (AVI 466 kb)
