# Supplementary material for: The bud tip is the cellular hot spot of protein secretion in yeasts
Source: Appl Microbiol Biotechnol. 2016 Jun 23;100:8159–68. doi: 10.1007/s00253-016-7674-6 (PMC4989006; doi:10.1007/s00253-016-7674-6)
Supplement: Supplementary file 1 — (PDF 321 kb) [file 253_2016_7674_MOESM1_ESM.pdf]

The bud tip is the cellular hot spot of protein secretion in yeasts

Applied Microbiology and Biotechnology

Verena Puxbaum[1,2], Brigitte Gasser[1,2], Diethard Mattanovich[1,2]
[1] Department of Biotechnology, BOKU – University of Natural Resources and Life Sciences, Vienna, Austria.
[2] Austrian Centre of Industrial Biotechnology (ACIB GmbH), Vienna, Austria.
E-mail: diethard.mattanovich@boku.ac.at

Electronic Supplementary Material:

Online Resource 1:

Time-lapse experiment of *P. pastoris* expressing HSA-oxGFP. The fluorescent images of layer 12 are displayed at a frame rate of 6 frames per second. The ER, and HSA within the ER are inherited to the daughter cell.

Online Resource 2:

Time-lapse experiment of *P. pastoris* expressing HSA-oxGFP. The fluorescent images of layer 17 are displayed at a frame rate of 6 frames per second. In the presented layer the remaining ER in the mother cell is monitored.

Online Resource 3:

Long-term time-lapse experiment of *P. pastoris* expressing HSA-oxGFP. Cells were embedded on a 4% agarose pad and monitored for 3 hours in a confocal laser scanning microscope. The fluorescent images of the most significant layer are presented at a frame rate of 2 frames per second.

Online Resource 4:

Long-term time-lapse experiment of *P. pastoris* expressing HSA-oxGFP. Cells were embedded on a 4% agarose pad and monitored for 3 hours in a confocal laser scanning microscope. The bright field images of the most significant layer are presented at a frame rate of 2 frames per second.

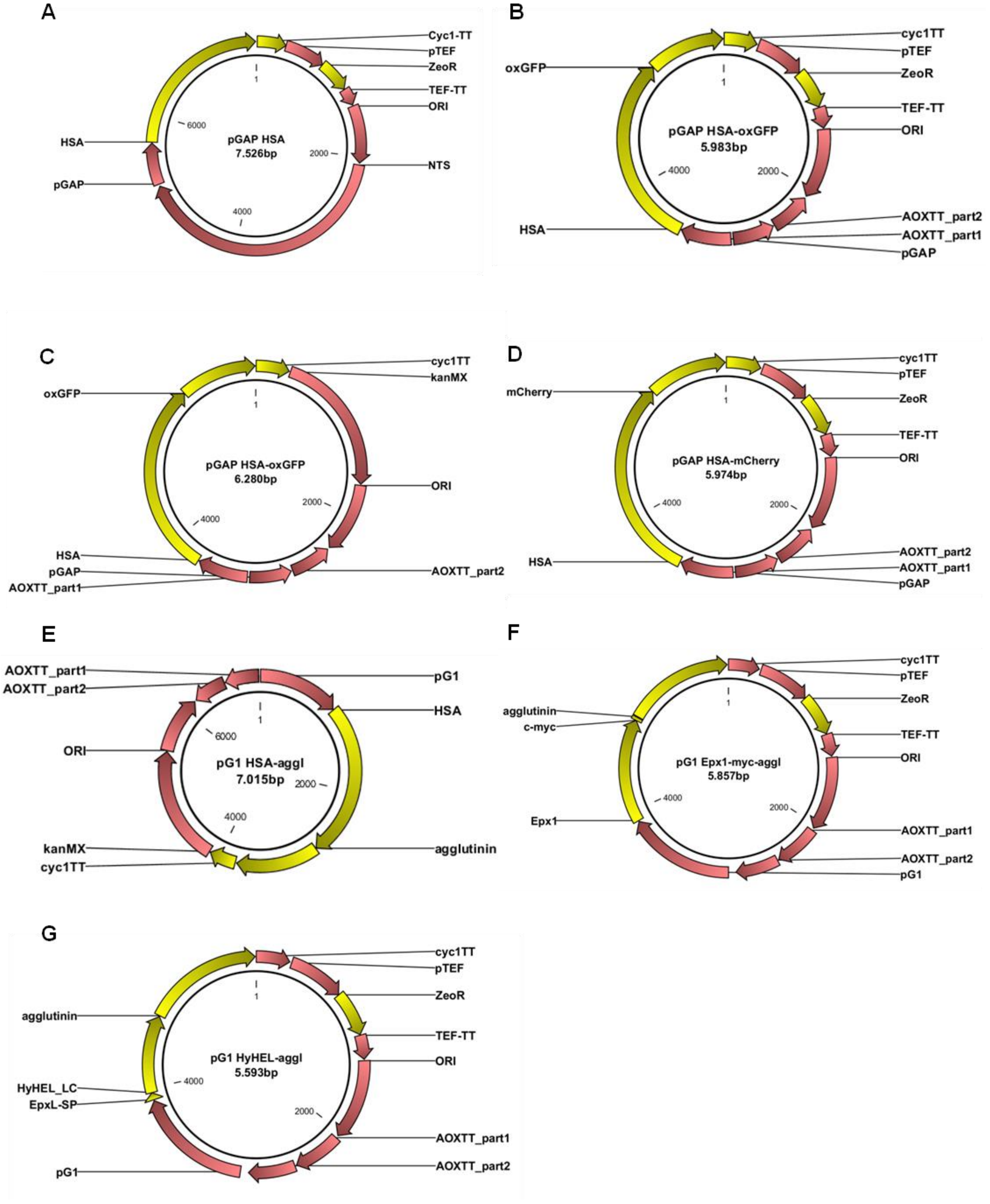

Fig. S1: Vector maps of plasmids used in this study. (A) pGAP HSA with zeocin resistance cassette. (B) pGAP HSA-oxGFP with zeocin resistance cassette. (C) pGAP HSA-oxGFP with kanamycin/G418

resistance cassette. (D) pGAP HSA-mCherry with zeocin resistance cassette. (E) pG1 HSA-agglutinin with kanamycin resistance cassette. (F) pG1 Epx1-c-myc-agglutinin with zeocin resistance cassette. (G) pG1ExpL-SP (signal peptide) HyHEL-LC-agglutinin with zeocin resistance cassette
